# Storage-dependent changes in the soluble urinary proteome: a pre-analytical stability study in inflammatory clinical urine samples

**DOI:** 10.1016/j.jlb.2026.100505

**Published:** 2026-09-21

**Authors:** Angelo Novak, Stephanie Tautges, Lisa Geissler, Florian Wagenlehner, Daniel R. Engel, Hannah Voss, Olga Shevchuk

**Affiliations:** a Department of Immunodynamics, Institute for Experimental Immunology and Imaging, University Hospital Essen, Essen, Germany; b Clinic for Urology, Pediatric Urology and Andrology, Justus-Liebig-University Giessen, Giessen, Germany

**Keywords:** Urine proteomics, Pre-analytical variability, Storage conditions, Proteome stability, Pyelonephritis, Mass spectrometry

## Abstract

Urine is a highly accessible biofluid for clinical proteomics, but pre-analytical variability during collection, storage, and processing can compromise reproducibility. Mass spectrometry–based proteomics enables broad measurement of urinary proteins, making it suitable for evaluating how storage conditions affect proteome composition. However, clinical translation of urinary proteomics is limited by insufficient standardization of pre-analytical workflows and limited knowledge of proteome stability during storage, compromising reproducibility. This limitation is particularly relevant for inflammatory urine samples, where immune-cell-derived and complement-associated proteins may continue to change ex vivo during delayed processing.

Here, we systematically evaluated the stability of the soluble urinary proteome under clinically relevant storage conditions. Urine samples from patients with pyelonephritis were used as a clinically relevant inflammatory urine model and processed under six pre-analytical storage conditions, including immediate freezing at −80 °C, repeated freeze–thaw cycles, and incubation for 30 min or 3 h at either 4 °C or room temperature prior to freezing. High-resolution proteomics using data-independent acquisition coupled with parallel accumulation–serial fragmentation (DIA-PASEF) identified more than 4,300 protein groups across four pyelonephritis patients.

Global proteome profiles were highly reproducible across all conditions (Pearson r > 0.9). In contrast, specific immune and metabolic proteins exhibited storage-dependent abundance changes, particularly after prolonged room temperature exposure, consistent with ex vivo immune activation. These results support standardized urine handling, particularly avoidance of prolonged room-temperature storage, to reduce storage-induced changes in immune-associated urinary proteins.

## Graphical abstract

Schematic overview of the workflow for the differential quantitative proteome analysis of the soluble urine fraction from pyelonephritis patients under different storage conditions. D, immediate freezing at −80 °C; F, three freeze–thaw cycles; RT, room temperature; LC-MS/MS, liquid chromatography–tandem mass spectrometry.

**Figure:**
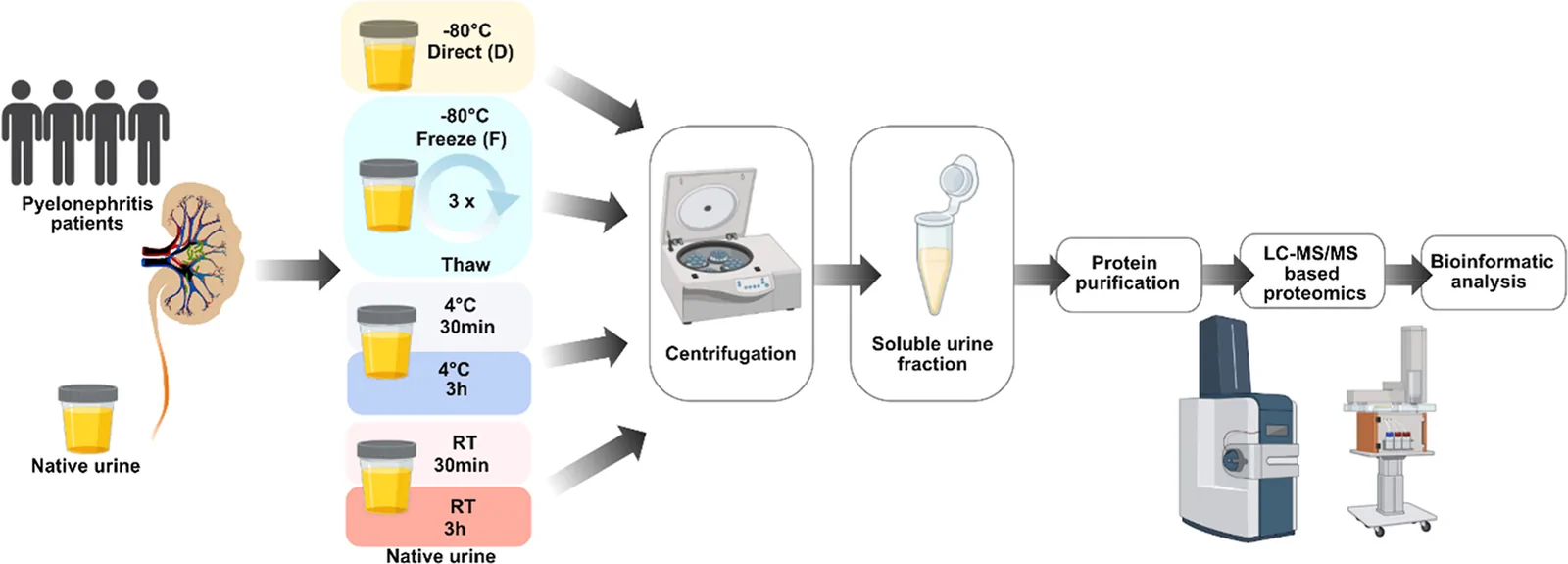

## 1 Introduction

Urine is an accessible and non-invasive biofluid for proteomic analysis, but reliable interpretation of urinary proteome data depends on standardized pre-analytical handling. Among various biofluids available for liquid biopsy, urine has emerged as a highly promising yet still underutilized sample, despite its considerable advantages over more established sources such as blood, saliva, gastric juice, sweat, tears and cerebrospinal fluid [1]. The unique accessibility, large obtainable volume, and non-invasive collection make urine particularly attractive for repeated sampling and longitudinal disease monitoring [2].

Because healthy kidneys filter vast amounts of plasma—about 150–180 L daily—urine contains proteins from two main sources: predominantly the urinary tract (about 70%) and, to a lesser extent, the bloodstream. This dual contribution makes urine a rich matrix for detecting both urogenital and systemic biomarkers [3]. Additionally, unlike plasma, which maintains homeostatic protein concentrations through sophisticated regulatory mechanisms, urine serves as a waste repository that accumulates changes over time, making it particularly sensitive to early disease-associated alterations that might be effectively masked in blood by homeostatic processes [1]. Moreover, from an analytical standpoint, the composition of urine proteome is less complex than that of serum or plasma, as it is less dominated by high-abundance proteins such as albumin and immunoglobulins that can interfere with analytical procedures [4].

Urinary proteomics has been increasingly applied to kidney and urogenital diseases, but its reproducibility depends strongly on sample collection, storage, and processing. Recent advancements in mass spectrometry-based urinary proteomics now allow the identification and quantification of thousands of proteins from individual urine samples. Improvements in sample preparation, chromatographic separation, and highly sensitive liquid chromatography–mass spectrometry (LC-MS) instrumentation enable a deep proteome coverage, high reproducibility across diverse LC-MS platforms and a high concordance of disease-specific urinary proteome signatures [5]. Together, these advances make large-scale urine proteomics feasible, increasing the need for robust pre-analytical workflows that preserve biologically meaningful protein patterns and support the discovery of new biomarkers for personalized medicine [5].

Urinary tract infections (UTIs) are among the most common bacterial infections worldwide and can lead to serious complications such as renal damage or urosepsis. Despite their high prevalence, the mechanisms driving UTI onset, persistence, and recurrence remain insufficiently understood, underscoring the need for improved diagnostic and monitoring strategies [6].

Routine urinary proteome profiling will only be reliable if storage-induced protein changes can be distinguished from true biological variation. A major barrier to the broader clinical adoption of urine proteomics is the incomplete understanding of proteome stability under routine storage conditions encountered in clinical practice. Unlike plasma proteomics, where standardized protocols have been extensively validated across platforms and laboratories, urine protein analysis still lacks robust, widely accepted guidelines for sample collection, storage, and processing that would ensure reproducible and clinically meaningful results [7].

This issue is critical due to urine's variable nature, the fact that bacterial and immune cell activity can markedly impact urinary proteomic and metabolomic profiles, and analysing urine is complicated by fluctuations in its ionic strength, pH, and osmolality influenced by factors like stress, nutrition, medications, health conditions, and environmental exposure. Current data on urinary protein stability and standardized preparation remain limited and often inconsistent [8]. For instance, extracellular vesicles in urine have been found to stay largely intact, with results showing they remain relatively stable for up to 6 h at 25 °C and up to 12 h at 4 °C [9]. In contrast, earlier studies show that while the overall urinary proteome is relatively stable during short-term storage, the urinary peptidome is considerably more susceptible to degradation, especially when processing is delayed or samples are kept at room temperature. The inclusion of protease inhibitors had a variable effect depending on the individual sample, highlighting the importance of standardized sample handling to ensure reproducibility in urinary proteomics and peptidomics analyses [10]. Additionally, a comprehensive review of urinary biomarkers used in cancer diagnostics further highlighted that many studies omit key information about pre-analytical conditions, despite growing evidence that proper sample handling is crucial for reproducibility. This gap undermines the reliability and potential of urinary proteomics across various diseases [7]. Therefore, investigation by Liu et al. contributes to the broader effort of standardizing urine proteomics methodologies across different analytical platforms and laboratory settings, addressing the critical need for harmonized protocols that enable data comparability and facilitate multi-centre collaborative studies [11].

This study addresses the pre-analytical stability of the soluble urinary proteome under clinically relevant storage conditions. Urine samples from patients with pyelonephritis (PN) were selected as an inflammatory clinical model because it contains immune-cell-derived and infection-related proteins that may be particularly vulnerable to ex vivo changes. The aim of this study was to determine which components of the soluble urinary proteome remain stable and which are affected by delayed sample processing, storage temperature, or repeated freeze–thaw exposure.

## 2 Materials and methods

### 2.1 Publicly available datasets

Public urinary proteome datasets were used to benchmark the depth and coverage of the present soluble urine dataset against recent urine proteomics studies. The following data sets were used: diabetic kidney disease (DKD): from Yun et al. [12], chronic kidney disease (CKD) from Khoza et al. [13] and snakebite-associated acute kidney injury (AKI) from Brasileiro-Martins et al. [14]. Because pre-analytical handling and storage workflows differ between studies and are not always fully reported, this cross-study comparison is intended to provide contextual insight into proteome depth and disease-associated signatures rather than direct quantitative comparison.

### 2.2 Urine samples from patients with PN

Urine was collected from acute PN patients and healthy control donors by the Clinic for Urology, Pediatric Urology and Andrology of the Justus-Liebig-University Giessen, Germany. The ethical approval has been received by the independent ethics commission of the University Giessen, Germany (Ethical vote 280/20) on January 6th, 2021. The cases of acute PN were clinically diagnosed, following the United States Food and Drug Administration (FDA) definitions used for studies in the indication "Developing drugs for treatment. Guidance for industry, June 2018" (https://www.fda.gov/media/71313/download). Patients admitted to the urological department at the University Hospital Giessen with at least two of the following signs or symptoms were included: chills, rigors, or warmth associated with fever; flank pain; nausea or vomiting; dysuria; urinary frequency; urinary urgency; costo-vertebral angle tenderness upon physical examination; or a urine specimen with evidence of pyuria. The cohort was established to investigate the pre-analytical stability of the urinary proteome rather than to identify biomarkers of pyelonephritis. Therefore, paired aliquots from each urine sample were subjected to different storage conditions, allowing the assessment of storage-induced condition.

### 2.3 Incubation conditions

To assess the proteome stability of the soluble urine fraction during storage, including the effect of potential ex vivo activated immune cells, 400 μl of native urine was either immediately frozen at −80 °C, or incubated at 4 °C or room temperature (RT) for 30 min or 3 h. After incubation, all samples were immediately frozen at −80 °C. To simulate multiple freezing-thaw cycles, samples were thawed on ice and re-frozen at −80 °C for three consecutive cycles.

### 2.4 Protein extraction and proteolytic digestion

After incubation, native urine samples were thawed on ice and centrifuged at 5,000 × g for 10 min at 4 °C. The supernatant (soluble urine fraction) was used for all subsequent analysis. Soluble proteins were extracted using acetone precipitation according to Pichler et al. [15]. Briefly, one volume of the soluble urine fraction was precipitated with four volumes of cold 100% acetone at −20 °C overnight, followed by centrifugation at 12,000 × g for 40 min at 4 °C. The pellet was washed twice with 1,000 μL of ice-cold 80% acetone, each wash performed for 5 min at 12,000 × g and 4 °C. The resulting protein pellet was resuspended in 8 M urea and 100 mM Tris(hydroxymethyl)aminomethane (Tris). For protein concentration estimation, samples were diluted to a final urea concentration of 1.6 M using LC-MS grade water. Protein concentrations were determined with the Pierce™ BCA Protein Assay Kit following the manufacturer's instructions. Twenty micrograms of protein were used for tryptic digestion. Samples were diluted to a final urea concentration of 2 M in 50 mM ammonium bicarbonate. Disulfide bonds were reduced with 5 mM dithiothreitol (DTT) for 30 min at 56 °C and alkylated with 20 mM iodoacetamide (IAA) for 30 min at RT in the dark. The reaction was quenched by the addition of 2.5 mM DTT. Proteolytic digestion was performed with the lysine-specific endoproteinase Lys-C (1:50 enzyme: substrate ratio) for 2 h at 37 °C, followed by trypsin (1:50 enzyme: substrate ratio) digestion overnight at 37 °C. Digestion was terminated by the addition of trifluoroacetic acid (TFA) to a final concentration of 1%. The resulting tryptic peptides were dried in a SpeedVac vacuum concentrator, desalted using Pierce^TM^ C18 Spin Tips & Columns (Thermo Fisher Scientific, Waltham, USA) according to the manufacture's instructions and dissolved in 40 μl of 0.1% formic acid (FA).

### 2.5 LC-MS/MS data acquisition

Prior to injection, the peptide concentration was estimated using a NanoDrop Microvolume Spectrometer (Thermo Fisher Scientific, Waltham, USA) at 205 nm. 300 ng of peptides were loaded onto an Evotip (Evosep Biosystems, Odense, Denmark) following the manufacturer's instructions. Briefly, Evotips were rinsed with 0.1% FA in acetonitrile (ACN), conditioned with 2-propanol, equilibrated with 0.1% formic acid, and then loaded using centrifugal force at 800g. Evotips were subsequently washed with 0.1% formic acid, and then 100 μL of 0.1% formic acid was added to each tip to prevent drying.

Peptide separation was performed on an Evosep One (Evosep Biosystems, Odense, Denmark) using the predefined thirty samples per day (30 SPD) method, on a PepSep Series column (C18, 15 cm length x 150 μm inner diameter, 1,5 μm particle size (Bruker Daltonics, Brillerica, Massachusetts, USA)).

Eluting peptides were injected into a TimsTOF HT mass spectrometer (Bruker Daltronics, Billerica, Massachusetts, USA), via a CaptiveSpray source at 1600V. The mass spectrometer was operated in data-independent acquisition coupled with parallel accumulation–serial fragmentatio (DIA-PASEF) mode. The accumulation and ramp time for the dual Trapped Ion Mobility Spectrometry (TIMS) analyzer were set to 100 ms at a ramp rate of 9.42 Hz. Scan ranges were set to mass-to-charge ratio (*m*/*z*) 100–1,700 for the mass range and inverse reduced ion mobility (1/K_0_) 0.60–1.60Vs-1cm-2 for the mobility range at both MS levels. For DIA-PASEF, a cycle time of 1.8 s was estimated. Within each cycle, a precursor mass range of 400 to 1201 Da and a mobility range of 0.60 to 1.60 1/K0 was fragmented. At precursor-ion (MS1) level, one ramp was executed per cycle. For fragment-ion (MS/MS) measurements 32 MS/MS windows with a mass width of 26.0 Da each and a mass overlap of 1.0 were distributed across the 16 MS/MS ramps.

### 2.6 Data processing

LC-MS raw data were processed using the direct-DIA algorithm in Spectronaut (Version 19.1.240806.626, Biognosys, Schlieren, Switzerland), applying Biognosys factory settings (Enzyme/Cleavage rules: Trypsin/P; Fixed modification: Carbamidomethyl (C); Variable modifications: Acetyl (Protein N-term) and Oxidation(M)), against a reviewed human protein sequence database from UniProtKB/Swiss-Prot in FASTA format (downloaded on 13th of March 2024, containing 20418 target sequences). Quantification was performed at the MS/MS level. Imputation and cross run normalization were disabled.

### 2.7 Statistical analysis

Unnormalized protein abundances were used for further downstream analysis, exported, and analysed in the R software environment (v 4.1.2; R Core Team 2021).

Protein abundances were log_2_ transformed to approach the Gaussian probability distribution. Data normalization to compensate for injection amount discrepancies was achieved by subtracting the mean abundance per sample for each protein quantified in the respective sample. Linearity of protein abundances between conditions per patient was assessed through the Pearson correlation. Prior to imputation, the probability distribution across all patients was tested using an Anderson-Darling test separately for each patient. Proteins with a p-value > 0.05 were considered Gauss distributed. For Gauss distribution-based imputation, at least 75% of all quantified proteins were expected to be normally distributed. Imputation was performed under the missing not at random (MNAR) hypothesis. Missing values were imputed from the normal distribution per protein, with a downshift of 1.3, toggling values across a range of 0.3 for each patient separately. Differentially abundant proteins between multiple predefined phenotypes were determined through analysis of variance (ANOVA). Proteins identified with a p-value < 0.05 were considered significant and subjected to further analysis.

To identify enriched pathways among differentially regulated proteins, REACTOME -based enrichment analysis was performed, using the Search Tool for the Retrieval of Interacting Genes/Proteins (STRING) database [16]. For all enrichment analysis gene sets identified as enriched with an adjusted false discovery rate (FDR) (Benjamini-Hochberg) < 0.05 were considered significantly enriched.

Heatmaps were generated based on hierarchical clustering results. The Pearson correlation was used as a distance metric at the sample and protein level, respectively. Ward. D2 linkage was applied. Prior to heatmap generation, log2 protein abundances were centered around the mean per protein for visual purposes. For principal component analysis (PCA), the first two principal components (PCs) were visualized as a scatter plot. To assess the similarity between groups from PCA, the top 3 PCs, representing at least 50% of the explained variance, were selected and averaged per group. The Euclidean distance between respective group values was calculated.

Within the R software environment, the following packages were used: ***openxlsx*** (Schauberger P, Walker A (2025). openxlsx: Read, Write and Edit xlsx Files. R package version 4.2.8, https://github.com/ycphs/openxlsx), ***dplyr*** (Wickham H, François R, Henry L, Müller K, Vaughan D (2025). dplyr: A Grammar of Data Manipulation. R package version 1.1.4, https://dplyr.tidyverse.org.), ***gridExtra*** (Baptiste Auguie (2015). gridExtra: Miscellaneous Functions for "Grid" Graphics. R package version 2.0.0. https://CRAN.R-project.org/package=gridExtra.),***ggplot2***, ***tidyr*** (Wickham H, Vaughan D, Girlich M (2025). *tidyr: Tidy Messy Data*. R package version 1.3.1, https://tidyr.tidyverse.org***.), UpsetR*** [17]***, mixOmics*** [18]***, naniar, lubridate, pheatmap*** (Kolde R (2025). ***pheatmap:*** *Pretty Heatmaps*. R package version 1.0.13, https://github.com/raivokolde/pheatmap.), ***RColorBrewer*** (Neuwirth E (2022). *RColorBrewer: ColorBrewer Palettes*. R package version 1.1-3, https://CRAN.R-project.org/package=RColorBrewer.), ***rstatix*** (Kassambara A (2023). rstatix: Pipe-Friendly Framework for Basic Statistical Tests. R package version 0.7.2, https://rpkgs.datanovia.com/rstatix/.), ***purr*** (Wickham H, Henry L (2025). *purrr: Functional Programming Tools*. R package version 1.1.0, https://purrr.tidyverse.org/.), ***knitr*** (Xie Y (2025). knitr: A General-Purpose Package for Dynamic Report Generation in R. R package version 1.50, https://yihui.org/knitr/.), ***stringr*** (Wickham H (2023). *stringr: Simple, Consistent Wrappers for Common String Operations*. R package version 1.5.1, https://github.com/tidyverse/stringr, https://stringr.tidyverse.org) ***nortest (***Gross J, Ligges U (2015). *nortest: Tests for Normality (*https://cran.r-project.org/web/packages/nortest/index.html*).* ***Corrplot (***Wei T, Simko V (2024). R package *'corrplot': Visualization of a Correlation Matrix* (Version 0.95), https://github.com/taiyun/corrplot.).

## 3 Results

To investigate urine proteome stability under clinically relevant conditions, we analysed samples from four patients with PN, a disease context characterised by immune cell infiltration, antimicrobial peptide release, and bacterial activity. We first evaluated whether the soluble urine workflow provided sufficient proteome depth. Public urinary proteome datasets were included only as a qualitative reference for proteome coverage and were not interpreted as a disease-comparison analysis. Across four PN patients, a total of 4,328 protein groups were identified. When compared to recent, technically comparable state-of-the-art proteomic studies on the soluble urine fraction from patients with non-infectious kidney disease - such as diabetic kidney disease (1,877 proteins), chronic kidney disease (1,469 proteins), acute kidney injury (2,168 proteins) -and from healthy donors (2,724 proteins). The present dataset contained 1,334 proteins not reported in the selected public urinary proteome datasets. (Fig. 1A, Supplementary Table 1). This supported the depth of the present dataset for stability analysis.

**Fig. 1:**
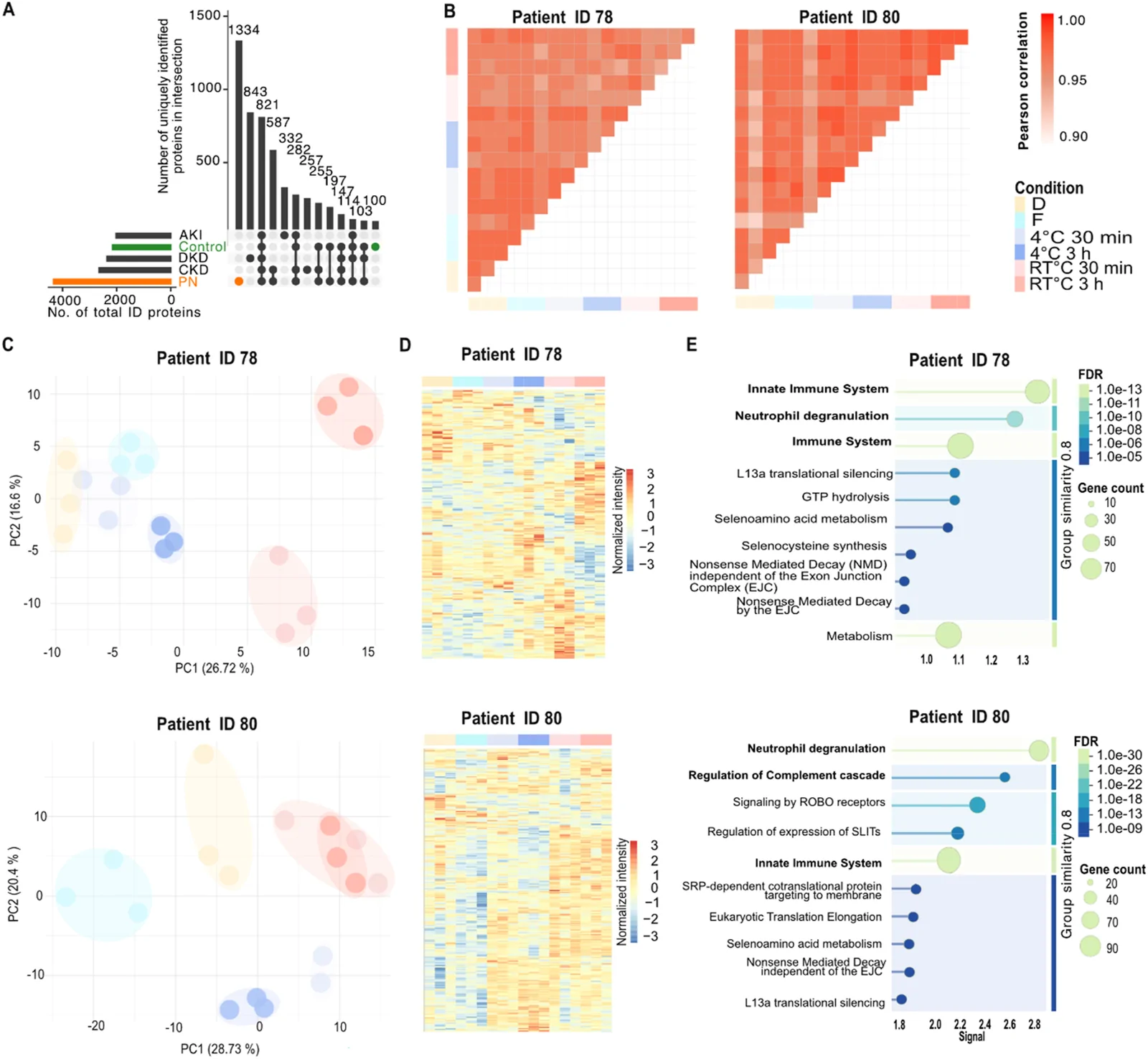
**Storage-dependent proteomic changes in urine of PN patients.** A) Upset plot overlaying proteins identified from the soluble urine fraction of common kidney diseases patients and healthy donors (chronic kidney disease (CKD); diabetic kidney disease (DKD); acute kidney injury (AKI); pyelonephritis (PN). B) Pearson correlation between replicates and storage conditions for exemplary patients. Correlations were calculated using log2 transformed, normalized data. C) Supervised principal component analysis (PCA) based on ANOVA significant proteins between all tested storage conditions. D) Heatmap visualization of Pearson correlation-based hierarchical clustering for all ANOVA significant proteins per patient. For better visualization, data points were centered across rows, prior to clustering. E) Signal-strength-based Top 10 enriched terms in REACTOME based enrichment analysis (q-value (Benjamini-Hochberg) < 0.01), based on ANOVA significant proteins per patient.

To assess the impact of pre-analytical storage conditions on the stability of the soluble PN proteome, patient samples were either directly frozen at −80 °C, subjected to three freeze–thaw cycles or incubated for 30 min or 3 h at 4 °C or room temperature (RT) prior to processing (see graphical abstract). At the global level, quantitative proteome patterns were highly reproducible, with Pearson correlations consistently exceeding 0.90 across all replicates and storage conditions. (Fig. 1B, Supplementary Fig. 1A).

Further analysis, however, revealed storage-dependent alterations affecting defined subsets of the urinary proteome. Across all patients, reproducible and statistically significant abundance changes were identified for 1,062 proteins across storage conditions (ANOVA, p < 0.05; Supplementary Table S2–S5). Principal component analysis of these proteins demonstrated distinct clustering according to storage condition, with directly processed samples and those immediately frozen clustering closely together. Prolonged storage at 4 °C and RT, and particularly 3h at RT resulted in the largest separation shift along the first principal component (Fig. 1C). Quantitative assessment of dissimilarities supports this observation, as Euclidean distances between frozen and direct samples were minimal, whereas samples stored for 3h at RT exhibited the greatest divergence on average (Supplementary Fig. 2).

Heatmap visualization of ANOVA-significant proteins further illustrated consistent differences between stable samples (directly processed or frozen), samples subjected to repeated freeze–thaw cycles, and those stored at 4 °C or RT, with the most pronounced shifts seen after prolonged storage at RT (Fig. 1D). Taken together, these results demonstrate that although the urinary proteome is globally stable, a subset of proteins undergo condition-dependent changes, particularly under prolonged RT exposure. REACTOME-based pathway enrichment showed that storage-sensitive proteins were predominantly associated with immunological processes, including “Innate immune system”, “Neutrophil degranulation” and “complement activation” (Fig. 1E, Supplementary Fig. 1D, Supplementary Table S6–S9), indicating that key infection-related immune effector mechanisms are particularly susceptible to pre-analytical handling. To identify the storage-dependent alterations of specific proteins, the significantly regulated proteins involved in “Neutrophil degranulation” and “complement cascade” were further analysed. The analysis revealed the increase of specific protein abundance after storage at 4 °C but especially RT (Fig. 2A, Supplementary Fig. 3C–E; Fig. 2B, Supplementary Fig. 3A and B). These data indicate an ex vivo activation of immune cells and the complement cascade during storage conditions, distorting immunological proteome profiles of the soluble urine fraction. Among others, the neutrophil-derived protein cathepsin G (Fig. 2C, Supplementary Fig. 4A), remained stable in directly analysed and repeatedly frozen but increased abundance after prolonged storage, particularly at RT. Similarly, the complement components C3 and C1R (Fig. 2 D, E, Supplementary Fig. 4B and C) were largely unaffected in frozen samples or samples stored short-term at 4 °C, yet exhibited pronounced abundance increases after 3h at RT and, to a lesser extent, after prolonged storage at 4 °C. These consistent trend across multiple patients underscore immune-associated proteins were among the most storage-sensitive proteins under delayed non-frozen handling.

**Fig. 2:**
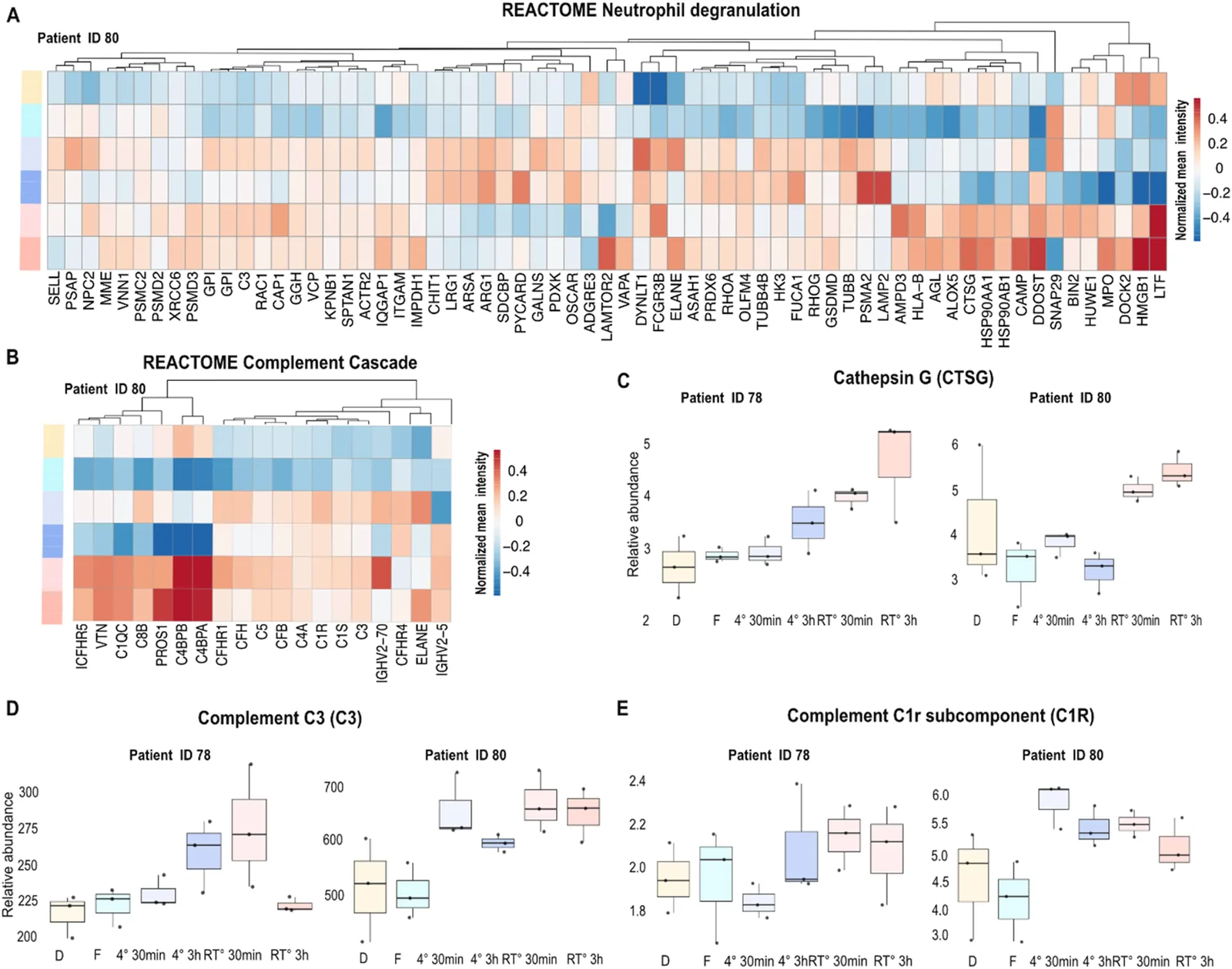
**Neutrophil degranulation and complement activation during storage at 4° and RT.** A) Heatmap visualization of the mean centered protein abundance per storage condition for ANOVA significant (p-value < 0.05) proteins associated with the REACTOME gene set “Neutrophil degranulation” for an exemplary patient. B) Heatmap visualization of the mean centered protein abundance per storage condition for ANOVA significant proteins associated with the REACTOME gene set “Complement cascade” in a selected patient. C-E) Relative protein abundance distribution for selected proteins affiliated with the REACTOME gene sets “Neutrophil degranulation” and “Complement cascade” across different storage conditions for exemplary patients. Normalized, imputed abundances were reverse log2 transformed and plotted.

## 4 Discussion

In this study, we systematically assessed the stability of the urinary proteome from PN patients under different storage conditions. These findings could be interpreted within the broader kidney disease proteomics landscape. Our results reveal a dual perspective: while the urinary proteome is remarkably stable at the global level, defined subsets of proteins—particularly those linked to immune effector pathways and cellular metabolism—undergo reproducible alterations during non-frozen storage conditions. These findings highlight both the strengths and limitations of urine as a liquid biopsy matrix and provide important guidance for pre-analytical handling.

A first key observation concerns the depth of proteome coverage achieved in the present soluble urine dataset. Across four patients, more than 4,300 proteins were identified, exceeding coverage reported in recent state-of-the-art urine proteome studies from non-infectious kidney diseases [[12], [13], [14]]. This observation is consistent with previous reports indicating that the urinary proteome reflects kidney- and urogenital-specific processes rather than representing a simple dilution of the plasma proteome [19]. Over 1,100 proteins were unique to PN, with strong enrichment for urogenital, urinary-tract, immune, and leukocyte-associated processes. Notably, all 16 antimicrobial peptides (AMPs) previously described in PN kidney tissue, as well as 33 of 34 reported neutrophil degranulation factors, were detected in the soluble urine fraction, indicating that the urine dataset contained immune and host-defense-associated proteins expected in inflammatory urinary samples [20]. Comparative analysis with urinary proteomes from diabetic kidney disease, chronic kidney disease, and acute kidney injury cohorts further illustrates this dual utility: PN urine overlaps with other kidney pathologies in common injury pathways, yet it also contains a distinct set of proteins enriched for urogenital and immune processes. This infection-specific signature, consistent with the pathophysiology of PN, forms coherent STRING clusters and underscores why inflammatory urine is a useful model for testing whether immune-associated proteins are vulnerable to pre-analytical handling [21,22].

We next evaluated the impact of storage conditions. Urine is an attractive clinical biopsy because it is non-invasive and easy to obtain. However, in routine clinical practice and biobanking workflows, samples are often stored for several hours before processing. At the global level, proteome-wide patterns were highly reproducible, indicating that urine proteomics is well suited for large-scale studies and biobank collections where perfect standardization is not always feasible.

More detailed analysis, however, revealed reproducible changes affecting a subset of storage-sensitive proteins, particularly after prolonged exposure at RT. Pathway enrichment of these proteins showed strong associations with neutrophil degranulation, neutrophil extracellular trap (NET) formation, complement and coagulation cascades. These findings are biologically plausible in the context of PN, where neutrophils and complement proteins are abundant in urine and can remain active ex vivo [23]. For example, neutrophil-derived proteins such as azurocidin (AZU1) and cathepsin G (CTSG) contribute to antimicrobial defence and inflammation-associated tissue remodelling, while complement factors C3 and C1R are central to innate immune activation [24,25]. Crucially, these proteins were stable under frozen or short-term conditions but shifted reproducibly after 3h at RT. The observation indicates that immune effector proteins—often considered candidate biomarkers—are particularly labile and susceptible to pre-analytical artifacts [26].

These insights parallel observations from other liquid biopsy studies. Plasma and serum proteomics, for example, are influenced by pre-analytical conditions, with clotting- and immune-related proteins showing particular sensitivity [27]. Our results extend this principle to urine, suggesting that immune pathways may represent a general class of “unstable” proteomic modules across biofluids. Recognizing and accounting for these vulnerabilities is essential to prevent false-positive biomarker associations and to ensure reproducibility in clinical proteomics.

From a translational perspective, these results have two key implications. First, the global urinary proteome was relatively robust across the tested handling conditions, supporting the feasibility of urine proteomics in studies where minor handling variation is unavoidable. Second, immune-related proteins arguably among the most clinically relevant for infection and inflammatory kidney disease are disproportionately affected by pre-analytical handling. To mitigate this, best practices should include immediate freezing at −80 °C, aliquoting to avoid repeated freeze–thaw cycles, using protein low-binding plastic to minimize adsorption. When feasible, centrifugation or filtration at the time of collection may further reduce ex vivo activity by removing intact cells. If immediate freezing is not possible, short-term storage at 4 °C is preferable to RT, but prolonged delays should still be minimized.

In summary, PN urine contains a rich and disease-specific proteomic signature that combines general kidney pathology with infection-related immune processes. While the urinary proteome is globally stable, subsets of immune and metabolic proteins are highly sensitive to pre-analytical handling, particularly prolonged storage at RT. Recognizing and controlling these vulnerabilities is critical for accurate biomarker discovery and clinical translation. By combining biological insight with practical recommendations, this work lays a foundation for the reliable use of urine as a liquid biopsy material in infection-focused and kidney disease research.

## Ethics declaration

Written informed consent to take part in the study and to publish the article has been obtained from all participants or their legal representatives. The privacy rights of participants have been observed.

This study included organ or tissue donors. This study includes human biological material and consent was obtained by donors, or their next of kin or legal representatives, for use in this study and for publication of the article. The samples used in this research were not sourced from executed prisoners or prisoners of conscience.

This study was performed in compliance with relevant laws, regulatory frameworks and guidelines where the research took place. This study was approved by the Urine was collected from acute PN patients and healthy control donors by the Clinic for Urology, Pediatric Urology and Andrology of the Justus-Liebig-University Giessen, Germany. The ethical approval has been received by the independent ethics commission of the University Giessen, Germany (Ethical vote 280/20) on January 6th, 2021. (Approval No. Ethical vote 280/20)

## Data sharing statement

LC-MS/MS raw data can be accessed through the ProteomeXchange Consortium (http://proteomecentral.proteomexchange.org) via the Proteomics Identifications Database (PRIDE) partner repository using the following reference: 1-20251126-141923-8657088 (Control donors, soluble urine proteome) and 1-20251125-114647-8657088 (PN patients, soluble urine proteome).

## Author contribution statement

AN, HV, FW, DRE, and OS conceptualized the study. AN, LG, and ST prepared and analysed the samples by LC–MS/MS. AN, OS, and HV performed the bioinformatic data analysis and visualization, and wrote the manuscript. All authors critically reviewed and approved the final manuscript.

## Funding

We received funding from the 10.13039/501100001659German Research Foundation: FOR5427/1-(466687329): FOR5427 SP1 (OS; FW) and FOR5427 SP4 (DRE). DRE received funding from the 10.13039/501100001659German Research Foundation: EN984/18-1 and 19-1; TRR332 A3 and Z1; INST 20876/486-1.

## Declaration of competing interest

The authors declare that they have no known competing financial interests or personal relationships that could have appeared to influence the work reported in this paper.
