# Supplementary material for: Storage-dependent changes in the soluble urinary proteome: a pre-analytical stability study in inflammatory clinical urine samples
Source: J Liq Biopsy. 2026 Sep 21;14:100505. doi: 10.1016/j.jlb.2026.100505 (PMC13635551; doi:10.1016/j.jlb.2026.100505)
Supplement: Supplementary file 1 [file mmc10.docx]

SUPPLEMENTARY INFORMATION

**Supplementary Figure 1:** A) Pearson correlation between replicates and storage conditions for each exemplary patient. Correlations were calculated using log2 transformed, normalized data. B) Supervised principal component analysis (PCA) based on ANOVA significant proteins between all tested storage conditions C) Heatmap visualisation of Pearson correlation-based hierarchical clustering for all ANOVA significant proteins per patient. For better visualisation, data points were centred across rows, prior to clustering. D) Signal strength-based Top 10 enriched terms in REACTOME based enrichment analysis (q-value (Benjamini-Hochberg) < 0.01), based on ANOVA significant proteins per patient.

**Supplementary Figure 2:** Euclidean distance in PCA, based on all PCs, reflecting at least the highest 50% of the explained variance.

**Supplementary Figure 3:** A-E) Heatmap visualization of the mean centered protein abundance per storage condition for ANOVA significant (p-value < 0.05) proteins associated with the REACTOME gene sets “Neutrophil degranulation” or “Complement cascade” for exemplary patients.

**Supplementary Figure 4:** A-C) Relative protein abundance distribution for selected proteins affiliated with the REACTOME gene sets “Neutrophil degranulation” and “Complement cascade” across different storage conditions for exemplary patients. Normalized, imputed abundances were reverse log2 transformed and plotted.
